# Circulating tumor DNA as a biomarker for progression and survival in esophageal cancer after neoadjuvant therapy and esophagectomy: a systematic review and meta-analysis

**DOI:** 10.1097/JS9.0000000000003017

**Published:** 2025-08-12

**Authors:** Tianzheng Shen, Tiancheng Li, Yuqin Cao, Yajie Zhang, Hecheng Li

**Affiliations:** Department of Thoracic Surgery, Ruijin Hospital, Shanghai Jiao Tong University School of Medicine, Shanghai, China

**Keywords:** circulating tumor DNA (ctDNA), esophageal cancer, neoadjuvant therapy (NAT), prognostic biomarker, survival outcomes

## Abstract

**Background:**

Circulating tumor DNA (ctDNA) has emerged as a promising biomarker for monitoring treatment response and predicting outcomes in cancer. This meta-analysis aimed to evaluate the prognostic value of ctDNA in predicting survival outcomes and pathologic complete response (pCR) in esophageal cancer (EC) patients undergoing neoadjuvant therapy (NAT).

**Methods:**

A systematic search of PubMed, Web of Science, and Embase was conducted between August and September 2024. Observational studies and randomized clinical trials were included if they reported associations between ctDNA detection and progression-free survival (PFS), overall survival (OS), or pCR. Fixed-effects models were used to pool hazard ratios (HRs) and odds ratios (ORs). Heterogeneity was quantified using *I*^2^ statistics, and publication bias was assessed via funnel plots and Egger’s test.

**Results:**

Among 71 studies screened, 10 met the inclusion criteria (*n* = 334 patients). Baseline ctDNA detection was not significantly associated with PFS, OS, or pCR (*P* > 0.05). However, ctDNA detection post-NAT was significantly associated with worse PFS (HR 3.81; 95% CI: 2.19–6.64), OS (HR 3.00; 95% CI: 1.64–5.50), and lower odds of pCR (OR 0.26; 95% CI: 0.09–0.73). Similarly, ctDNA detection post-surgery predicted worse PFS (HR 4.17; 95% CI: 2.17–8.04) and OS (HR 4.00; 95% CI: 1.90–8.42).

**Conclusion:**

ctDNA detection following NAT and surgery is a strong predictor of poor survival outcomes and lower pCR rates in EC. These findings underscore ctDNA’s potential as a biomarker for risk stratification and personalized treatment planning in EC patients. Further standardization of ctDNA detection methods is essential to optimize its clinical utility.

## Introduction

Esophageal cancer (EC) is among the leading causes of cancer-related mortality globally^[1]^, being particularly prone to early latent metastasis and poor prognosis. Despite advances in treatment, most EC patients present at locally advanced stages. Neoadjuvant therapy (NAT), including chemoradiotherapy (NCRT), chemotherapy (NCT), immunotherapy (NIT), and combinations thereof (NICT/NICRT), has been increasingly applied for locally advanced resectable EC, as evidenced by the survival benefits shown in trials in both adenocarcinoma^[2,3]^ and squamous cell carcinoma^[4]^. However, due to the high heterogeneity of EC, predicting response to NAT remains a significant challenge.

**HIGHLIGHTS**

- This is the first meta-analysis to systematically evaluate ctDNA’s prognostic value in esophageal cancer patients after neoadjuvant therapy, incorporating data from classical and immunotherapy-based NAT regimens.
- Post-treatment ctDNA positivity (detected in 13–20% of patients) should trigger intensified surveillance/adjuvant therapy due to 3- to 4-fold higher progression/death risk.
- Current heterogeneity in detection methods (dPCR/NGS/sWGS) precludes universal ctDNA thresholds; standardization is urgently needed for clinical adoption.

Circulating tumor DNA (ctDNA), a non-invasive biomarker derived from tumor cells, has gained attention for its role in detecting minimal residual disease (MRD), which can lead to recurrence and metastasis in various cancers^[5–8]^, and shown emerging potential as a complementary tool to tissue-based diagnostics in EC^[9]^. ctDNA detection following NAT and surgery has been explored in several retrospective studies^[10,11]^, which have demonstrated associations between post-treatment ctDNA and prognosis. Although ctDNA has shown promise in monitoring treatment response in EC^[12,13]^, no standardized method for its analysis currently exists, and robust evidence linking ctDNA dynamics to prognosis in NAT-treated EC patients is lacking. Past studies have mainly focused on the diagnostic accuracy of ctDNA in EC or patients receiving direct surgery without NAT^[14,15]^, highlighting its potential in EC management. However, with the advancement of clinical trials and the updating of clinical guidelines, there is still insufficient evidence across all NAT phases (from baseline to post-operation) or accounting for modern immunotherapy regimens. The prognostic utility of ctDNA for EC patients undergoing NAT remains undefined and requires further validation.

To address this gap, we conducted a systematic review and meta-analysis pooling data from 10 studies (*n* = 514) to investigate the prognostic value of ctDNA in patients with EC receiving NAT. This study evaluates ctDNA’s prognostic value at multiple timepoints and includes recent trials with immunotherapy-based NAT, aiming to provide evidence-based guidance for its clinical application^[16]^.

## Methods

### Study design

A systematic review of the literature and meta-analysis according to the Preferred Reporting Items for Systematic Reviews and Meta-Analysis (PRISMA) guideline^[17]^ was conducted (Supplemental Digital Content Material, available at: http://links.lww.com/JS9/E867), to identify studies that assess the association of the presence of ctDNA and clinical outcomes in EC patients eligible for NAT. The work has been reported in line with AMSTAR (Assessing the methodological quality of systematic reviews) Guidelines (Supplemental Digital Content Material, available at: http://links.lww.com/JS9/E867)^[18]^. The meta-analysis is registered in the International Prospective Register of Systematic Reviews (PROSPERO 2024). PubMed, Web of Science, and Embase were searched by two independent authors between August and September 2024. Eligible studies were selected based on predefined criteria, with discrepancies resolved through discussion with a third author.

### Study selection

The primary endpoint of the meta-analysis was to investigate the impact of ctDNA detection on PFS and OS. Secondary endpoints included the association of ctDNA detection with pathologic complete response (pCR). ctDNA detection served as the predictor variable for these outcomes.

To be eligible for inclusion, the studies had to satisfy the following criteria: (i) observational studies (prospective or retrospective) and randomized controlled trials, (ii) human studies involving EC patients undergoing NAT, and (iii) documented serial collection of ctDNA and outcome data such as pCR, progression-free survival (PFS), disease-free survival (DFS), relapse-free survival (RFS), and overall survival (OS). The follow-up duration for PFS and OS varied among the included studies. We extracted and summarized the median follow-up time reported in each study, which is presented in Table 1. These follow-up durations ranged from 15 to 37 months, providing a reasonable timeframe to assess survival outcomes across studies. All methods like digital PCR (dPCR)^[10]^, next-generation sequencing (NGS) gene panels, and shallow whole-genome sequencing (sWGS) of ctDNA detection and analysis were allowed, given the lack of a gold standard and of direct comparisons between the various methods. Exclusion criteria included: (i) review articles, editorials, comments, and letters to the editor, and (ii) ongoing studies with results not presented or published at the time of the literature search. Moreover, studies were excluded if they reported incomplete data after scrutinizing available supplementary data. The authors and institutions were reviewed to avoid repetition of databases. The search strategy is detailed in Supplemental Digital Content Material, available at: http://links.lww.com/JS9/E867.

**Table 1:** Characteristics of included articles (*N* = 514)

Study	Cancer subtype	Type of neoadjuvant therapy administered	Whether adjuvant therapy administered	Number of patients underwent ctDNA test	Duration of follow-up	Sample collection	Method used to detect ctDNA	Site of mutations
Cabalag 2022	EAC	NCRT	No	55	15.2 months	Blood and tissue samples	NGS gene panels	APC, AR1D1A, CDKN2A, KRAS, NRG1, P1K3CA, SMAD4, SMARCA4, TP53
Kelly 2024	EAC = 28 (81%) ESCC = 6 (19%)	NCRT + immuno	No	32	36.4 months	Blood and tissue samples	NGS gene panels	TP53(31%), ALK(11%), BRCA2(9%), ERBB2(6%), ATM(6%), NTRK1 (4%), KRAS (3%), EGFR(2%), PIK3CA(2%)
Morimoto 2023	ESCC	NCT	Optional	16	17.5 months	Blood and tissue samples	NGS gene panels	TP53 (85%), CDKN2A (*n* = 1), NFE2L2 (*n* = 1)
Ng 2023	ESCC	NCRT	Optional	35	24 months	Blood samples	NGS gene panels and dPCR	TP53 (62.6%), NFE2L2 (15.4%), PIK3CA (11.4%)
Ococks 2021	EAC	NCT	Yes	97	32.9 months	Blood samples	NGS gene panels	TP53 (12.15%), APC (6.8%), KRAS
van den Ende 2023	EAC	NCRT	Optional	103	22 months	Blood samples	sWGS	TP53 (72%), KRAS (13%), CDKN2A (11%)
Yue 2024	ESCC	NCRT + immuno	Yes	38	17 months	Blood and tissue samples	NGS gene panels	NA
Hofste 2022	EAC = 68 (87%) ESCC = 8 (9%)	NCRT	No	78	30 months	Blood and tissue samples	NGS gene panels	TP53 (60%)
Wang 2022	ESCC	NCRT	No	40	20.6 months	Blood and tissue samples	NGS gene panels	TP53 (85.7%), PRSS3 (21.4%), CDKN2A (17.9%), ART (14.3%), PIK3CA (14.3%)
Verschoor 2024	EAC	NCT + immuno	Optional	20	37 months	Blood and tissue samples	WES	NA

EAC, Esophageal Adenocarcinoma; ESCC = Esophageal Squamous Cell Carcinoma; NCRT, Neoadjuvant Chemoradiotherapy; NCT, Neoadjuvant Chemotherapy

The methodological quality of the studies was assessed by two authors independently using the Reporting Recommendations for Tumor Marker Prognostic Studies (REMARK) checklist^[19]^. Discrepancies were resolved by consensus or by a third author. The REMARK score was used as a descriptive parameter of study quality, and no studies were excluded on this basis. The risk of bias of eligible studies was assessed comprehensively according to the Cochrane Collaboration’s Risk of Bias tool and the assessment of Risk Of Bias In Systematic reviews tool (ROBIS)^[20,21]^.

### PICO framework

Population (P): Patients with EC undergoing NAT (of any type) followed by surgery.
Intervention (I): Detection of ctDNA at baseline, after NAT, and after surgery.
Comparison (C): ctDNA-negative vs. ctDNA-positive status.
Outcomes (O): PFS, OS, and pCR.

### Data extraction

The following variables were collected from the selected publications and related published supplementary data: author, year of publication, type of NAT administered, EC subtype, number of patients undergoing ctDNA tests, method of ctDNA analysis, information regarding pCR, PFS (composite endpoint including major pathological response, RFS, cancer specific survival, and DFS, depending on the study), and OS. In cases where hazard ratios (HRs) and corresponding 95% confidence intervals (CIs) were not directly reported, survival data were extracted from Kaplan–Meier (K-M) curves. High-resolution images of K-M curves were obtained from the original articles, and survival probabilities were traced by marking data points corresponding to specific time intervals and their associated survival probabilities. The precision of the estimated HR is improved by extracting as many accurately positioned data points as possible across the curve. These time-survival data were then used to estimate log HRs and their variances following the method described by Parmar *et al*^[22]^. To summarize the overall effect, the odds ratio (OR) with 95% confidence intervals (CI) was calculated for the pCR analysis. For the PFS and OS analysis, the HR with 95% CI was calculated for each study to obtain an overall estimation. No p-values are reported for pooled HRs and ORs. Heterogeneity estimation was calculated and reported in all analyses (by means of *I*^2^ and a statistical test to evaluate the null hypothesis of homogeneity across studies). Fixed and random models were fitted regardless of the result of the homogeneity statistical test (when *P* > 0.05, the null hypothesis of homogeneity was not rejected). For the fixed effects analysis, the inverse variance method for pooling was used to calculate the overall HR assuming a common effect. Conversely, the random effects analysis was performed using the DerSimonian-Laird method to take heterogeneity into consideration. Funnel plot analysis and Egger’s test were performed to detect publication bias. All analyses were undertaken using R statistical software version 4.4.1 (R packages meta).

### Certainty of evidence

The certainty of evidence was evaluated using the GRADE framework. This approach assesses the quality of evidence across five domains: risk of bias, inconsistency, indirectness, imprecision, and publication bias. The evidence for each outcome was rated as high, moderate, low, or very low certainty. Detailed GRADE assessments are presented in Supplemental Digital Content Material, available at: http://links.lww.com/JS9/E867.

## Results

### Description of studies and patients

**Figure 1.**
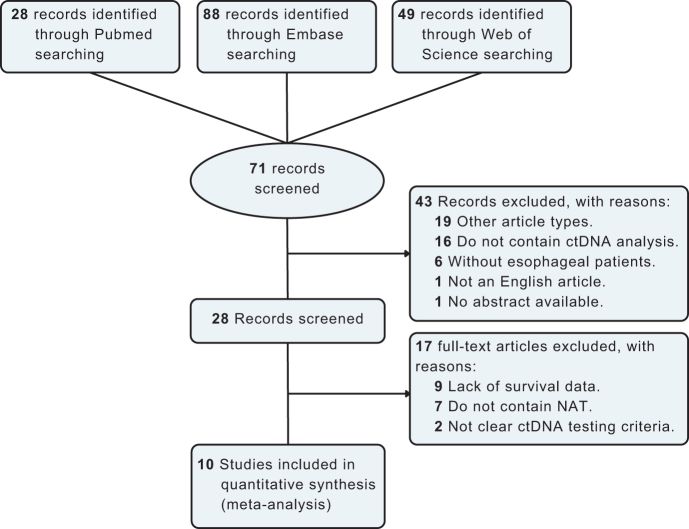
Flow diagram of article screening.

A total of 10 articles were finally selected for inclusion in the systematic review and meta-analysis according to PRISMA (Fig. 1, Table 1). Among the 10 studies, eight were observational studies with ctDNA collection at various timepoints^[11,23–29]^, and two were exploratory translational analyses of randomized clinical trials^[13,30]^. Regarding histological type, four studies included only EAC^[23,26,27,30]^, another four studies included only patients with ESCC^[11,25,28,29]^, rest two studies included patients with both EAC and ESCC^[13,24]^. All studies included NAT among which include five NCRT^[11,23,24,27,28]^, two neoadjuvant chemotherapy^[25,26]^, and three NCRT/NCT combined with immunotherapy^[13,29,30]^. Endpoint definitions of the included trials and the studies that were included in each endpoint of the meta-analysis can be found in Supplemental Digital Content Table 1, available at: http://links.lww.com/JS9/E865. Supplemental Digital Content Table 2, available at: http://links.lww.com/JS9/E866, describes the number of patients with evaluable blood samples and ctDNA detection at baseline, after NAT, and after surgery.

## Primary endpoint: association of ctDNA with PFS and OS

### ctDNA at baseline

Two studies (*n* = 130) reported data on baseline ctDNA detection and outcome^[24,28]^. Presence of ctDNA at baseline was identified in 73/118 (62%) of the patients who had received ctDNA tests included in the PFS analysis and OS analysis. Overall, the presence of ctDNA at baseline did not seem to influence the outcome. Positive results of ctDNA were not associated with a statistically significant worse PFS or OS (*P* > 0.05, Supplemental Digital Content Figure 1, available at: http://links.lww.com/JS9/E864). Importantly, medium heterogeneity was found across studies (*I*^2^ = 40%). The estimation of this analysis was performed using both the fixed effects model to account for the heterogeneity between studies.

### ctDNA after NAT

**Figure 2.**
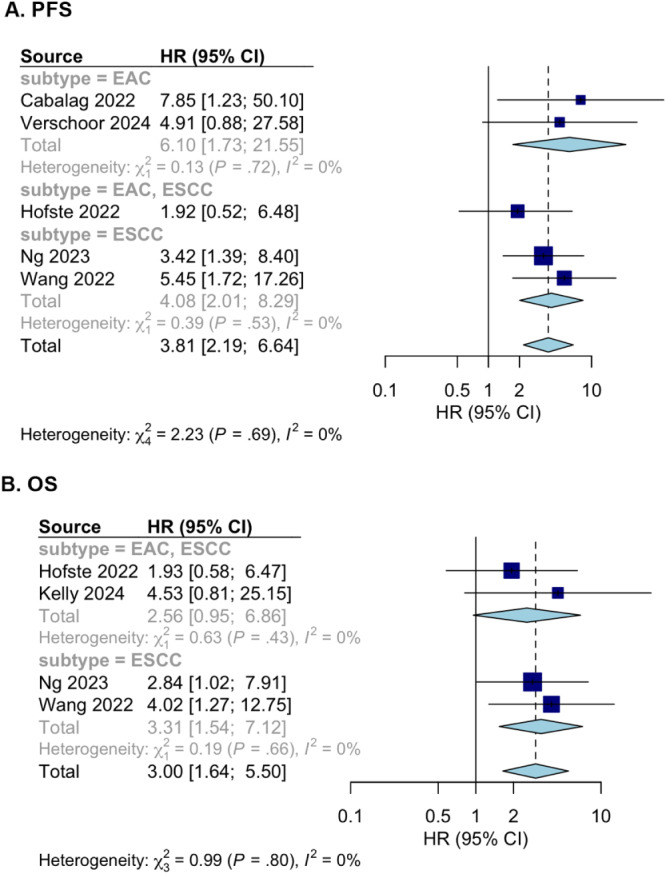
(A) Forest plot of the effect of ctDNA on OS after NAT (*n* = 206); (B) Forest plot of the effect of ctDNA on PFS after NAT (*n* = 194).

Five studies (*n* = 226) reported data on ctDNA detection after completion of NAT, among which three have reported both OS and PFS^[11,24,28]^, while the rest two studies have reported only OS^[13]^ or PFS^[30]^. ctDNA after completion of NAT was detected in 16/121 (13%) patients and associated with a statistically significant worse PFS (HR 3.81, 95% CI: 2.19-6.64, Figure 2A). ctDNA was found in 26/133 (20%) patients after NAT and associated with worse OS (HR 3.00, 95% CI: 1.64-5.50, Figure 2B). Due to the limited number of studies, subgroup analysis according to EC subtype was only feasible for ESCC. In ESCC^[11,28]^, persistence of ctDNA after NAT was found that significantly related to higher PFS (HR 4.08, 95% CI: 2.01–8.29) and OS (HR 3.31, 95% CI: 1.54–7.12).

### ctDNA after surgery

**Figure 3.**
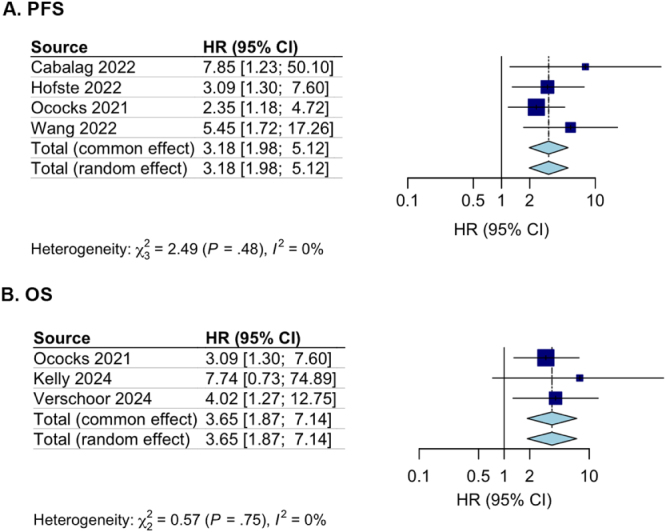
(A) Forest plot of the effect of ctDNA on OS after surgery (*n* = 149); (B) Forest plot of the effect of ctDNA on PFS after surgery (*n* = 185).

Six studies (*n* = 334) reported data on ctDNA detection after Surgery following NAT treatment. However, three have reported PFS^[23,24,28]^, the other three have only reported OS data^[13,26,30]^. ctDNA after completion of NAT and surgery was associated with a statistically significant worse PFS (HR 4.17, 95% CI: 2.17-8.04, Figure 3A) and OS (HR 4.00, 95% CI: 1.90–8.42, Figure 3B).

### Secondary endpoint: association of ctDNA with pCR

**Figure 4.**
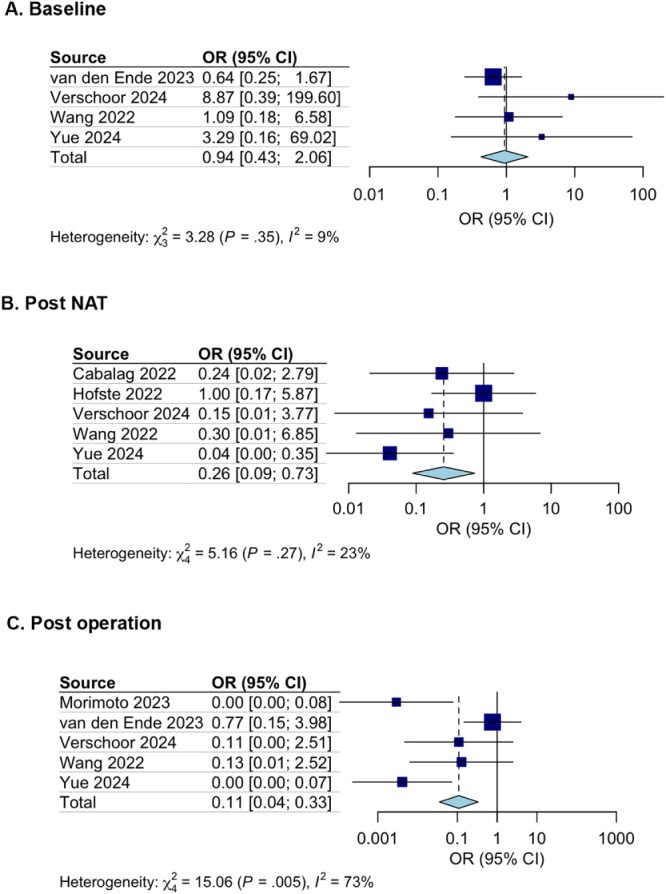
(A) Forest plot of the effect of ctDNA on pCR at baseline; (B) Forest plot of the effect of ctDNA on pCR after NAT; (C) Forest plot of the effect of ctDNA on pCR after surgery.

Four studies (*n* = 213) were combined for evaluation of the odds of achieving pCR if ctDNA was present at baseline^[21,22,29,30]^. Estimation with the fixed effects model showed no association between ctDNA status at baseline and pCR outcome (OR = 0.94; 95% CI: 0.43–2.06; Figure 4A). Five studies (*n* = 245) were combined for evaluation of the odds of achieving pCR if ctDNA was present after NAT^[23,24,28–30]^. Same as survival outcomes, estimation showed the presence of ctDNA status after NAT led to a worse pCR outcome (OR = 0.11, 95% CI: 0.04-0.33; Figure 4B). Nevertheless, evidence of heterogeneity was found across the OR estimation in the included studies (*I*^2^ = 73%). Five studies (*n* = 242) were analyzed for evaluation of the odds of achieving pCR if ctDNA tested positive after surgery (25, 27, 28, 29, 30). Results showed the presence of ctDNA status after surgery led to a worse pathological outcome (OR = 0.26; 95% CI: 0.09–0.073; Figure 4C).

### Quality assessment and risk of bias analysis

**Figure 5.**
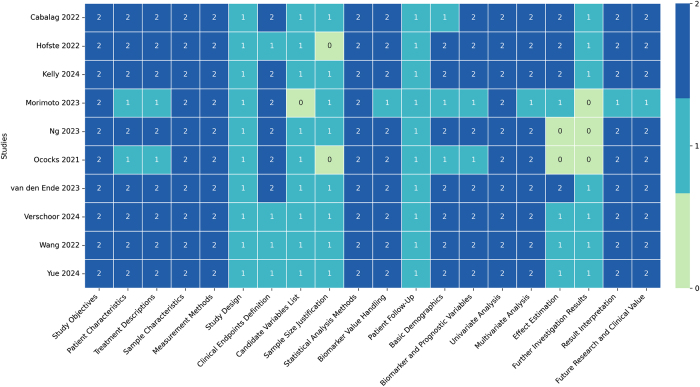
Heatmap of REMARK tools scores.

All studies were scored according to REMARK with a range of 24 to 35, with 40 being the maximum possible score (Fig. 5). Risks of publication bias are graphically summarized in a funnel plot in Supplemental Digital Content Figure 2, available at: http://links.lww.com/JS9/E864.

## Discussion

This meta-analysis provides several insights into the prognostic significance of ctDNA in EC patients undergoing NAT. Our findings highlight that ctDNA detection, particularly after NAT and surgery, is significantly associated with worse PFS and OS, as well as lower rates of pCR. These results suggest that ctDNA status, assessed as key treatment milestones, could serve as an essential biomarker for risk stratification and personalized treatment planning in EC.

Several studies have explored the potential of ctDNA as a biomarker in various cancers, including breast^[31]^, colorectal^[7]^, and lung cancers, with a growing number of evidence showing its potential utility in monitoring treatment response, detecting MRD, and predicting recurrence. However, unlike in breast cancer, where baseline ctDNA levels have been shown to correlate with outcomes^[31]^, our results indicate that ctDNA detection at baseline in EC patients does not have a significant association with PFS, OS, or pCR. This diverse may be attributable to the unique tumor biology and heterogeneity of EC, suggesting that the therapeutic effect of NAT on esophageal tumors is optimistic and the timing of ctDNA assessment plays a crucial role in its prognostic utility.

The association of ctDNA with survival outcomes likely reflects its correlation with tumor burden and shedding; persistent ctDNA may indicate ongoing microscopic disease or an aggressive tumor phenotype^[32,33]^. Additionally, ctDNA dynamics during NAT, such as early clearance or persistence, may offer valuable prognostic insights. Early ctDNA clearance could signify a favorable treatment response, while persistence may suggest residual disease and higher recurrence risk. However, due to the variability in assessment methods and time points across studies, our meta-analysis could not fully explore these dynamic changes, highlighting a crucial area for future research. Current studies rarely collect ctDNA samples during NAT, and when they do, the collection times vary between patients, limiting the ability to monitor treatment responses in real-time.

From a clinical perspective, the detection of ctDNA after NAT and surgery may provide relevant information for ameliorating treatment strategies. Patients who remain ctDNA-positive following NAT could be at a higher risk of recurrence and may benefit from additional therapeutic interventions. For example, if ctDNA levels suggest that a patient may not respond well to NCT, clinicians could consider adding immunotherapy or radiotherapy to enhance the therapeutic effect. The persistence of ctDNA in the postoperative period could indicate the presence of residual disease, prompting closer monitoring or consideration of adjuvant or salvage therapies. Instead, in patients who are ctDNA-negative post-treatment, de-escalation of therapy or surveillance-based strategies may be considered to avoid overtreatment. Incorporating ctDNA into the perioperative decision-making process could thus refine postoperative planning, enhance risk stratification, and support individualized treatment pathways in EC management. However, whether ctDNA dynamics correlate with radiologically assessed tumor response or tumor biology remains unclear. Although standard imaging was routinely used in many included studies to assess tumor response post-NAT, among the studies contained, few reported comparative data between imaging findings and ctDNA status. This limits our ability to evaluate the concordance between radiological and molecular assessments. Future studies that incorporate both imaging and ctDNA monitoring in a structured, time-coordinated fashion may provide a more comprehensive evaluation of tumor behavior and treatment efficacy.

Subgroup analyses in our study showed a strong association between ctDNA persistence post-NAT and poor survival outcomes in ESCC. These findings, derived from several observational studies, underscore the importance of ctDNA as a biomarker in ESCC. However, the limited number of studies on EAC prevents definitive conclusions regarding ctDNA’s prognostic value across different EC subtypes. This limitation highlights the need for future research to encompass a broader range of EC subtypes and conduct subtype-specific analyses to validate ctDNA’s prognostic implications in diverse patient populations. It is also worth noting that ctDNA levels released by local esophageal tumors are generally lower than those of other tumor types, and adenocarcinoma tends to release lower levels of ctDNA compared to squamous cell carcinoma^[34]^. This means that ctDNA can only be monitored in a subset of patients, limiting its utility as a universal biomarker. Therefore, it is necessary to combine ctDNA assessment with other approaches, such as radiological and pathological analysis, or adopt more sensitive detection techniques for patients with locally advanced EC.

Recent technological advances in ctDNA detection have expanded its clinical potential beyond mere presence or absence. Ultra-sensitive NGS platforms, including error-suppressed sequencing and personalized tumor-informed assays, now allow for the detection of low-frequency variants with high precision^[35]^. Emerging modalities such as ctDNA methylation profiling and fragment size analysis (fragmentomics) offer additional layers of biological information that may improve sensitivity and specificity in early disease detection and MRD assessment^[36]^.

Our study has several limitations. First, there was notable heterogeneity among the included studies, particularly in terms of ctDNA detection techniques, the timing of sample collection, and the types of neoadjuvant therapies administered. To mitigate these issues, we predefined ctDNA as a binary variable (positive vs. negative), which allowed us to harmonize and compare results across studies using different platforms and protocols. Second, while subgroup analyses were attempted where sufficient data permitted, the small number of studies using each specific method limited further stratification. Another important limitation is the use of composite endpoints (PFS, DFS, RFS) across the included studies, which may impact the interpretation of pooled results. Despite these limitations, our study highlights the significant potential of ctDNA as a biomarker for guiding personalized treatment strategies in EC. Future prospective studies and randomized clinical trials could be remindful on validating ctDNA’s role in risk stratification, particularly in identifying high-risk patients who may benefit from additional therapies post-NAT. Standardizing ctDNA detection methods and exploring the dynamics of ctDNA changes during treatment are also necessary to fully realize its clinical utility.

## Conclusion

This meta-analysis demonstrates that ctDNA status after NAT and surgery predicts survival and treatment response in EC, supporting its integration into risk-adapted strategies. Standardized protocols and prospective trials are needed to optimize its clinical utility.

## Data Availability

The data supporting the findings of this study are available from the corresponding author upon reasonable request. The datasets used and/or analyzed during the current study are derived from publicly available sources as cited in the manuscript or were generated during the study.

## Research registration unique identifying number (UIN)

PROSPERO 2024 CRD4202458174.
